# Interpreting trials on renal replacement therapy initiation: beware of methodologic issues

**DOI:** 10.1186/s13054-020-02961-z

**Published:** 2020-05-19

**Authors:** Stéphane Gaudry, Paul M. Palevsky, Didier Dreyfuss

**Affiliations:** 1 Département de réanimation médico-chirurgicale, APHP Hôpital Avicenne, Université Sorbonne Paris Nord, Bobigny, France; 2 Common and Rare Kidney Diseases, French National Institute of Health and Medical Research, INSERM UMR_S 1155, Sorbonne Université, Paris, France; 3 Health Care Simulation Center, UFR SMBH Université Sorbonne Paris Nord, Bobigny, France; 4 Renal Section, Medical Service, Veterans Affairs Pittsburgh Healthcare System, Pittsburgh, PA USA; 5 Renal-Electrolyte Division, Department of Medicine, University of Pittsburgh, Pittsburgh, PA USA; 6 AP-HP, Médecine Intensive-Réanimation, Hôpital Louis Mourier, 92700 Colombes, France; 7 Université de Paris, Paris, France; 8 Present address: Médecine Intensive-Réanimation, Hôpital Louis Mourier, 178 rue des Renouillers, 92110 Colombes, France

No large-scale randomized controlled trial (RCT) on the initiation strategy for renal replacement therapy (RRT) in critically ill patients with acute kidney injury (AKI) was available for years. Expert opinion [1] and consensus conferences [2] recommend a conservative approach with RRT initiated only when life-threatening complications (hyperkalemia, intractable acidosis, or diuretic-unresponsive pulmonary edema) are present. However, RRT is often initiated earlier based mainly on amount of hourly urine output and/or urea nitrogen or serum creatinine concentration [3] even in the absence of the abovementioned complications. This attitude is based on the putative deleterious effects of high levels of nitrogen waste products and of hypothetical ill-effects of mediators of inflammation or other elusive factors. This approach assumes that theoretical advantages of early RRT surpass its actual risks, including catheter-related problems, hypotension, complications of anticoagulation, and the risk that treatment may actually prolong the course of AKI.

For many years, only small RCTs [4] and observational studies of the timing of RRT [5] were available. Observational studies compared patients with AKI who received early RRT with those who received it later but did not account for patients who either recovered or died without receiving RRT [6]. These patients who did not require RRT during AKI were shown to have a good prognosis [7] and not including them biases analyses comparing early to delayed strategies. Not surprisingly, most meta-analyses concluded that “early” RRT was associated with better survival [8].

Only RCTs comparing strategies for RRT initiation could solve the conundrum [6] (Fig. 1). Patients should not be simply randomized to “early” or “late” RRT but rather to an early strategy in which patients with severe AKI receive RRT shortly after the diagnosis is made or to a delayed strategy in which RRT is postponed until specific criteria for initiation are met. One single-center [9] and two multicenter [10, 11] RCTs utilized such protocols. Their conclusions differed: the former reported lower mortality with the early strategy but the two others did not show any mortality difference. Our purpose is not to delineate which study is correct, although the former received several criticisms [12]. A recent individual patient-data meta-analysis of RCTs included more than 1500 patients and showed that mortality was not affected by the strategy of RRT initiation and that 42% of patients allocated to a delayed strategy did not receive RRT [13].

**Fig. 1:**
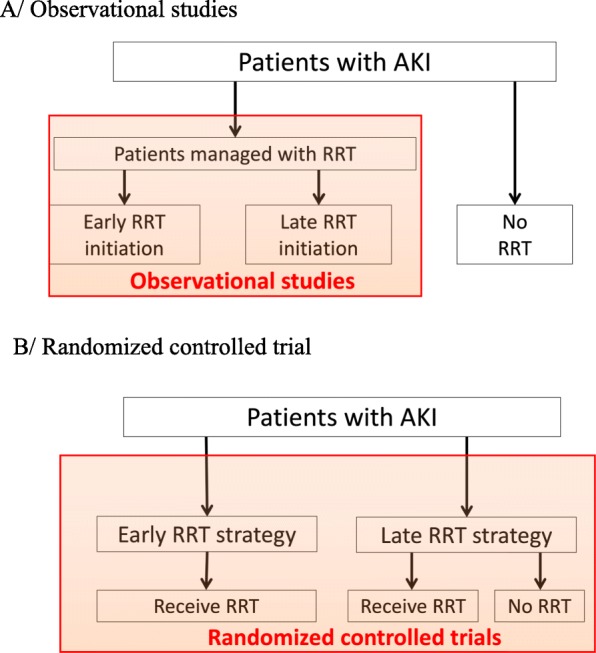
Different study designs to evaluate the timing of initiation of kidney replacement therapy in acute kidney injury. **a** Observational studies do not include patients with AKI who never receive RRT. **b** Randomized controlled trials adequately answer clinical question of timing of RRT in AKI. Not all patients included in the late initiation strategy actually will receive RRT. Adapted with permission from Palevsky et al. [6]

A methodological distortion would analyze patients based on post hoc groups by splitting the delayed strategy in two artificial subgroups: those who did or did not ultimately receive RRT. Such analyses will likely find that patients in the delayed arms who ultimately received RRT had worse outcomes than those who received early RRT. This falls back into the methodological flaws of prior observational studies. At the point of randomization, patients allocated to either the early or delayed strategies form a heterogeneous population. Some patients rapidly recover or die for reasons unrelated to their AKI and even less so to the assigned RRT strategy. Some have a more prolonged course during which they may suffer from complications unrelated to AKI. A delayed strategy allows time for clarifying the medical situation of individual patients. Thus, three disparate populations with differing mortality risk profiles may become apparent: those who recover kidney function without reaching specified objective criteria for RRT, those who die of non-renal complications prior to RRT initiation, and those with prolonged AKI, who ultimately meet the pre-specified criteria for RRT. The pseudo group of patients randomized to delayed RRT who ultimately receive RRT per protocol have time to develop complications in the ICU either because of the severity of their initial disease or because of additional events.

This flaw can be illustrated using two theoretical examples. First, a patient with a urinary tract infection and septic shock has KDIGO stage 3 AKI. Their hemodynamic condition rapidly improves, but oliguria persists for several hours, and they are allocated to an early RRT strategy and receive RRT rapidly. Their diuresis resumes within 24 h, and kidney function improves making further RRT unnecessary. They are alive after 60 days. Had this patient been allocated to the delayed group, it is unlikely that they would have received RRT. In addition, survival is not the result of “early” RRT initiation. In a second example, a patient with severe atherosclerosis has abdominal sepsis with septic shock. Anuria develops, and they are randomized to a “delayed RRT strategy”. At randomization they have no severe metabolic complication. After 2 days, their sepsis worsens and they develop hemodynamic instability requiring vasopressor administration. Persistence of anuria and development of hyperkalemia mandate RRT. Subsequently, they develops massive hemiplegia. A CT-scan shows cerebral infarction in the middle cerebral artery territory with massive edema which is rapidly complicated by cerebral herniation. Brain death ensues. These complications cannot reasonably be attributed to the strategy of delayed RRT initiation.

Subgroup analysis based on post-randomization disease trajectory and not on baseline variables is flawed. Indeed, this comparison is subject to two kinds of bias [14]. First, indication bias: by definition, patients who finally received RRT in the “delayed group” were those who needed it because of the unfavorable evolution of their AKI and overall illness. This results in time-varying confounding. Second, immortal time bias: since patients who received RRT in the delayed strategy did not receive it per randomized allocation, but as a result of follow-up.

Taken together there is a logical coherence between methodological and pragmatic arguments to demonstrate without any ambiguity that timing of RRT does not affect prognosis and that artificially creating groups based on results is a trap that the wise clinician should avoid.

Convincing clinicians that “less may be more” may be difficult (even if this leads to much less use of RRT and substantial savings). As stated by JPA Ioannidis [15]: “Yet, how likely is it that physicians will design studies whose results may threaten their jobs by suggesting that less procedures, testing, interventions are needed … … Is EBM doomed to be heartily accepted only when it leads to more medicine, even if this means less health?”

## Data Availability

Not applicable.

## Abbreviations

AKI: Acute kidney injury
RCT: Randomized controlled trial
RRT: Renal replacement therapy
ICU: Intensive care unit
EBM: Evidence-based medicine
